# Dosimetry and radioprotection evaluations of very high energy electron beams

**DOI:** 10.1038/s41598-021-99645-7

**Published:** 2021-10-12

**Authors:** Thongchai A. M. Masilela, Rachel Delorme, Yolanda Prezado

**Affiliations:** 1 Institut Curie, Université PSL, CNRS UMR3347, Inserm U1021, Signalisation radiobiologie et cancer, 91400 Orsay, France; 2 Université Paris-Saclay, CNRS UMR3347, Inserm U1021, Signalisation radiobiologie et cancer, 91400, Orsay France; 3 Univ. Grenoble Alpes, CNRS, Grenoble INP, LPSC-IN2P3, 38000 Grenoble, France

**Keywords:** Translational research, Biological physics

## Abstract

Very high energy electrons (VHEEs) represent a promising alternative for the treatment of deep-seated tumors over conventional radiotherapy (RT), owing to their favourable dosimetric characteristics. Given the high energy of the electrons, one of the concerns has been the production of photoneutrons. In this article we explore the consequence, in terms of neutron yield in a water phantom, of using a typical electron applicator in conjunction with a 2 GeV and 200 MeV VHEE beam. Additionally, we evaluate the resulting ambient neutron dose equivalent at various locations between the phantom and a concrete wall. Through Monte Carlo (MC) simulations it was found that an applicator acts to reduce the depth of the dose build-up region, giving rise to lower exit doses but higher entrance doses. Furthermore, neutrons are injected into the entrance region of the phantom. The highest dose equivalent found was approximately 1.7 mSv/Gy in the vicinity of the concrete wall. Nevertheless, we concluded that configurations of VHEEs studied in this article are similar to conventional proton therapy treatments in terms of their neutron yield and ambient dose equivalent. Therefore, a clinical implementation of VHEEs would likely not warrant additional radioprotection safeguards compared to conventional RT treatments.

## Introduction

Radiotherapy (RT) is based on the use of ionising radiation to inflict DNA damage on cancer cells, ultimately inducing tumoral cell death. It is often used in conjunction with chemotherapy in order to achieve better tumor control^1^, and approximately 50% of cancer patients are expected to receive a round of RT during the course of their cancer treatment, with some of the more common treatment modalities involving the use of photons, electrons, or protons^2^. Conventional treatments using electrons of 4 to 25 MeV can be used to treat superficial tumors due to the nature of their dose deposition in depth. Although these characteristics are well suited for these superficial tumors, their short penetration depth and significant lateral scattering make them unsuitable for the treatment of deep-seated tumors.

Historically, the collimation of electron beams has been carried out using a diaphragm and a cone or tube attachment, which is what is referred to nowadays as an electron applicator^3^. The use of these applicators in electron beam therapy has been standardised and is described in ICRU report 71^4^. The Varian 2300C/D, Siemens Primus, and Elekta SLi are some of the commonly used clinical accelerators for electron beam therapy, and their respective applicators are of the diaphragm type as described in the aforementioned report, with the Varian and Elekta variations having open sidewalls (i.e. no conical/tubular section) while the Siemens variation is only partially opened^5^. Despite the concerted effort to move towards multileaf collimator technology for low energy electron treatments, the standard practice in clinics is still to use patient-specific cut-outs placed in the insert tray at the end of an applicator to further conform the dose to the target^6^. These cut-outs are often made from a material known as Cerrobend. The dosimetric characteristics of these Cerrobend inserts has been compared to tubular applicators, and although similar PDDs (percentage depth dose) and lateral dose profiles are observed, the use of a tubular applicator yields a lower Bremsstrahlung contribution at higher energies while the use of a Cerrobend insert affords more flexibility in terms of conforming the beam to a specific shape^7^.

In contrast to low energy electrons, very high energy electron (VHEE) beams of 150 to 250 MeV have been proposed as an alternative treatment modality for deep-seated tumors owing to their various dosimetric advantages^8^. Among those advantages is the increased inertia of VHEEs, resulting in an increase in the practical range, and a narrowing of the beam penumbra at depth—both of which becomes more severe with increasing beam energy^8,9^. Resultingly, the dose distributions of VHEEs are favourable compared to those of photon beams. Furthermore, due to the absence of electronic disequilibrium at interfaces, VHEEs have been shown to be relatively insensitive to tissue heterogeneities^9,10^, experiencing a less than 15% dose deviation in the central plane of the beam compared to therapeutic proton and photon beams, which can experience a deviation of up to 100% and 74% respectively when cuboid inserts of 0.001–2.2 g/cm$^3$ are embedded in the water phantom^11^. Additionally, the capability of electrons to be electromagnetically scanned due to their charged nature opens up the possibility of their use in conjunction with spatially fractionated radiotherapy (SFRT) techniques^12,13^.

There has recently been a renewed interest in VHEEs due to the technological advancements of compact high-gradient RF-based accelerators and laser wakefield accelerators based on laser-plasma technology, which overcomes one of the limitations originally foreseen for VHEEs, namely the large size of the linear accelerator (LINAC) that would be needed for such high energy beams. Wakefield accelerator technologies provide not only a compact, cost-efficient alternative for the production of electron beams^14^, but have also been shown to be capable of producing dose distributions comparable to that of photon beams while exploiting the advantages linked with the delivery of electron beams, namely a more precise manipulation with fewer mechanical components and shorter, more intense electron bunches^15,16^. Another interesting advantage of these laser wakefield accelerators is their role in the delivery of FLASH (ultra high dose rate) irradiations. Given that the FLASH effect can only be exploited under certain combinations of beam parameters^17^, the flexibility of the modulation of beam parameters afforded by these types of accelerators makes FLASH-VHEE treatments an exciting prospect. Previous animal studies with low energy electrons^18,19^ have already illuminated the normal tissue sparing effects of FLASH, but the use of VHEEs could exploit the benefits of FLASH while also providing an enhanced depth of penetration.

There are, however, some limitations when it comes to the use of this wakefield accelerator technology. The first limitation, and one of the foremost problems currently facing this technology, is the control and stability of these beams. There are studies that are continually being carried out in an attempt to improve certain characteristics of these beams, such as its reproducibility and beam pointing uncertainty^20,21^. Additional physical collimation might therefore still prove necessary in order to better conform the dose to a target. Secondly, the high energy of these electrons is associated with high entrance and exit doses. One of the solutions to this problem was proposed by Kokurewicz et al., who showed that the use of a magnetic focusing lens placed around the patient enables highly localised dose deposition in a small volumetric element for electron beams of 200 MeV and 2 GeV^22^. Although the limitation of high entrance and exit doses can be overcome by magnetic focusing, this capability is out of reach for most clinical facilities. Additionally, the space requirements of scanning dipoles and quadrupoles may pose a logistical challenge in terms of the space constraints of a clinical setting, thus diminishing the advantages of the otherwise comparative compactness of the technology. Due to these aforementioned limitations, and given the fact that electron applicators are currently used in a clinical setting, we postulated that we might benefit from reduced beam penumbras with its use.

One of the concerns with VHEEs is the production of secondary neutrons due to their enhanced biological effectiveness. Between a specific threshold and approximately 30 MeV, neutrons will be produced in all materials due to the giant dipole resonance. This threshold is approximately 10 to 19 MeV for light nuclei and 4 to 6 MeV for heavy nuclei^23^. Therefore the high energy photons produced in collisional and radiative interactions both in a collimating structure and a patient’s body are potentially a concern, given that neutrons are primarily produced through the photonuclear reactions ($\gamma ,n$), ($\gamma ,p$), ($\gamma ,2n$), and ($\gamma ,pn$)^8,24^.

Consequently, through Monte Carlo (MC) simulations, we explored some of the characteristics of collimated and uncollimated VHEE beams, specifically a 2 GeV and 200 MeV beam, and analysed the potential impact of secondary neutrons arising in all cases. We calculated the dose profiles (both in depth and laterally), absorbed doses in a target, and the particle fluences within a water phantom. Despite previous studies having quantified the production of neutrons within the water phantom itself and determined it to be relatively low^8,22,24^, this study is differentiated by several factors which highlight the novelty of this work. Firstly, the energies we evaluated (up to 2 GeV) are much higher than some of these other studies and may therefore be more susceptible to the production of photoneutrons. Secondly, we have performed an investigation into the radioprotection considerations within a treatment room. The out-of-field ambient neutron dose equivalents were calculated in order to be able to better situate VHEE treatments amongst conventional photon and proton treatments in terms of the risk of secondary cancers. These dose equivalents were calculated at various locations in the ambient air surrounding a water phantom, and the contribution from a semi-infinite concrete wall was investigated. While the yield of neutrons in air in the immediate vicinity (less than 20 cm) of a water phantom has been previously evaluated^24^, this is the first theoretical study to look at distances of up to 3 m from the water phantom, evaluate the neutron contribution from a concrete wall at these distances, and compare the resulting dose equivalents to current conventional treatments. Furthermore, we explored the consequences that the presence/absence of an applicator has on the yield of neutrons, which has not yet been done. This was valuable as the use of an electron applicator in conjunction with VHEEs could possibly correspond to the upper limit, and worse case scenario in terms of neutron production with a treatment room.

## Materials and methods

### Monte Carlo simulations

All the simulations were performed using TOPAS^25,26^ version 3.5, which is a MC software that wraps around the Geant4 simulation toolkit, leveraging Geant4’s functionality through the use of a parameter control system. It was originally validated against proton therapy measurements from the MGH (Massachusetts General Hospital) beamline^27^, and has since been an active player in the benchmarking of Geant4^26^. Traditionally, the MC code MCNP (Monte Carlo N-Particle Transport) has been used for neutron/photon physics and the calculation of neutron yields for radioprotection purposes^28^, however some of the more modern codes such as Geant4 have the advantage of not only being open source, thus enabling more widespread use and cross-validation, but also being written in a modern computing language (C++). Furthermore, Geant4 has been extensively benchmarked in the field of medical physics^29,30^, and has been found to be comparable to another MC code (PENELOPE) for radiation shielding applications^31^. In terms of its applicability to VHEEs, TOPAS has been validated through comparisons to FLUKA, in which good agreement was found between the respective dose distributions and beam spread, thus making it a viable alternative to older MC codes in the study of VHEEs^32^. In accordance with the recommendation of AAPM TG-268^33^, the main characteristics of the MC simulations performed are summarised in Table 1, with further specifics provided in the sections which follow.

**Table 1:** Summary of the main characteristics of the MC simulations.

Item	Description
Code	TOPAS^25,26^ version 3.5. Released on the 21st of June 2020
Validation	Originally validated against proton therapy measurements from the MGH (Massachusetts General Hospital) beamline^27^. TOPAS is an active player in the benchmarking of Geant4^26^
Timing	Simulations were performed on the Joliot Curie-SKL computational cluster. CPUs: 2x24-cores Intel Skylake@2.7GHz (AVX512). A total of 10$^7$ primary histories were launched per simulation PDD curves: 3 cycles of 10 simulations. Approximately 205 h (2 GeV) and 25 h (200 MeV) of CPU time/cycle. Lateral dose profiles: 1 cycle of 10 simulations. Approximately 105 h (2 GeV) and 13 h (200 MeV) of CPU time/cycle Absorbed dose in target: 1 cycle of 10 simulations. Approximately 205 h (2 GeV) and 22.9 h (200 MeV) of CPU time/cycle Particle fluences in water phantom: 1 cycle of 10 simulations. Approximately 70 h (2 GeV) and 9 h (200 MeV) of CPU time/cycle Particle fluences in air: 1 cycle of 10 simulations. Approximately 386 h (2 GeV) and 42.7 h (200 MeV) of CPU time/cycle Ambient neutron dose equivalent: 1 cycle of 10 simulations. Approximately 376.7 h (2 GeV) and 43.9 h (200 MeV) of CPU time/cycle
Source description	Monoenergetic 2 GeV and 200 MeV electron beams were simulated Source 1: Gaussian source with a FWHM of 15.9 cm and a divergence of 5$^\circ$. Beam characteristics were taken from the FLUKA input files of Kokurewicz et al.^34^. This source was use for all simulations except the lateral dose profiles Source 2: Gaussian source with a FWHM of 1 cm and a divergence of 0.3$^\circ$. This source was used for all the lateral dose profile simulations
Cross-sections	Standard Geant4 physics cross section data files were used from the string model based reference physics list: QGSP_BERT_HP_EMZ^35^. All simulations barring the neutron dose equivalent simulations were repeated with the BIC and INCLXX models which took up largely the same amount of hours
Transport parameters	The cut for all particles was maintained at the default of 0.05 mm
Variance reduction	None
Scored quantities	The *DoseToMedium* discretized volume scorer was used for absorbed doses in PDDs, lateral dose profiles, and target doses. Surface scorers were utilised for fluences in the water phantom/ambient air, and the *AmbientDoseEquivalent* scorer was used to calculate the neutron dose equivalents. The dose equivalents reported in this work were obtained using the conversion coefficients of Pelliccioni^36^, and differences observed when using the coefficients of ICRU report 95^37^ are shown in the Supplementary Material
Statistical uncertainties	Statistical uncertainties (type A) were calculated by means of the history by history approach. Specific values are provided in the relevant sections in the text
Post-processing	The total absorbed dose in the target was normalised to 2 Gy (Fig. 3). This normalisation was carried out by calculating a scaling factor for which when multiplied with the total absorbed dose in the target, would yield 2 Gy. This factor was then applied to the different absorbed dose contributions of primary and secondary electrons, photons, positrons, and neutrons. Post-processing of remaining results involved the use of python scripts to effectively convert the raw data into graphs, however no other normalisation/filtering of the data was applied

The *QGSP_BERT_HP_EMZ* reference physics list was used for all simulations in this study. The hadronic options are specified by *QGSP* (Quark Gluon String model with the Precompound model used for nuclear de-excitation) and *BERT* (Bertini-style Cascade) for energies greater than 12 GeV and below 10 GeV respectively. Transitioning between the two models is handled by the *FTF* (Fritiof) model. Geant4 considers four types of neutron interactions: radiative capture, elastic and inelastic scattering, and fission. These interactions are handled by the *HP* (High Precision) neutron model. And finally, *EMZ* designates the electromagnetic physics options—in this case *emstandard_opt4*^35^. Both the *QGSP_BERT* and *QGSP_BIC* (Binary Cascade) physics lists are suitable for radiation protection and medical applications, however *BERT* is more suited to higher energies, while the *BIC* option is preferable for hadron therapy applications at energies below 200 MeV due to its increased accuracy around the Bragg peak^30,38^.

In TOPAS, the distribution of the quantity of interest is used to calculate the statistical uncertainty according to the numerically stable algorithm of Knuth^39^. The standard deviation of the mean/sum is found by dividing/multiplying by the square root of the total number of histories simulated^25,26^, therefore unless otherwise stated all statistical uncertainties mentioned in the text are standard errors. As highlighted in Table 1, a total of 100–300 million primary particles were simulated in batches of 10 million in order to reach a satisfactory compromise between computation time and accuracy. The standard errors were then computed by using the summation in quadrature method. These standard errors are therefore representative of type A uncertainties as they are a measure of the statistical uncertainty of the MC scoring whereas the type B uncertainties are defined as those evaluated by any other means^40^. Specifically for comparative MC studies, there could be inter-code differences which include but are not limited to, uncertainties in the inherent physics parameters—such as the interaction cross sections or models utilised, simulation geometries, and mass attenuation coefficients (which govern the particle transport)^41^. Given that one of the primary objectives of this study was the calculation of the neutron fluences and ambient neutron dose equivalents arising under various configurations, and given that a comparison of these dose equivalent values was carried out in Table 2 between values obtained herein and those obtained in other studies, we performed an estimation of the type B uncertainty involved in the production of neutrons and the resulting conversion into an ambient dose equivalent. Further details are provided below in the relevant sub-sections of the “Materials and methods” section. Ultimately, the type A and type B uncertainties were combined in quadrature as this provides an adequate estimation of the combined standard uncertainty for MC simulations^42^. The uncertainty associated with each dose equivalent value reported in this work was therefore obtained using the aforementioned summation.

#### Simulation geometry

Three configurations were considered: a collimated and uncollimated VHEE beam, both with an SSD (source to surface distance) of 100 cm, as well as an uncollimated beam with a reduced SSD of 5 cm. For the collimated configuration, a typical electron applicator as utilised in clinical electron beam therapy was placed between the source and the water phantom^43^. Aluminium (*G4_Al*) with a density of 2.699 g/cm$^3$, which is a predefined material from the Geant4 material database, was chosen as the material for this applicator. In accordance with the commonly used clinical accelerators referenced in the introduction section, an open sidewall diaphragm design was considered as opposed to a conical/tubular applicator. This necessitated the use of a Cerrobend block to further conform the beam to a desired shape. A 5 cm thick Cerrobend block with a density of 9.4 g/cm$^3$ and a material composition of 50% bismuth, 26.7% lead, 13.3% tin, and 10% cadmium^7^ was therefore placed in the insert tray at the end of the applicator, and which collimates the beam down to a 2 $\times$ 2 cm$^2$ field.

**Figure 1:**
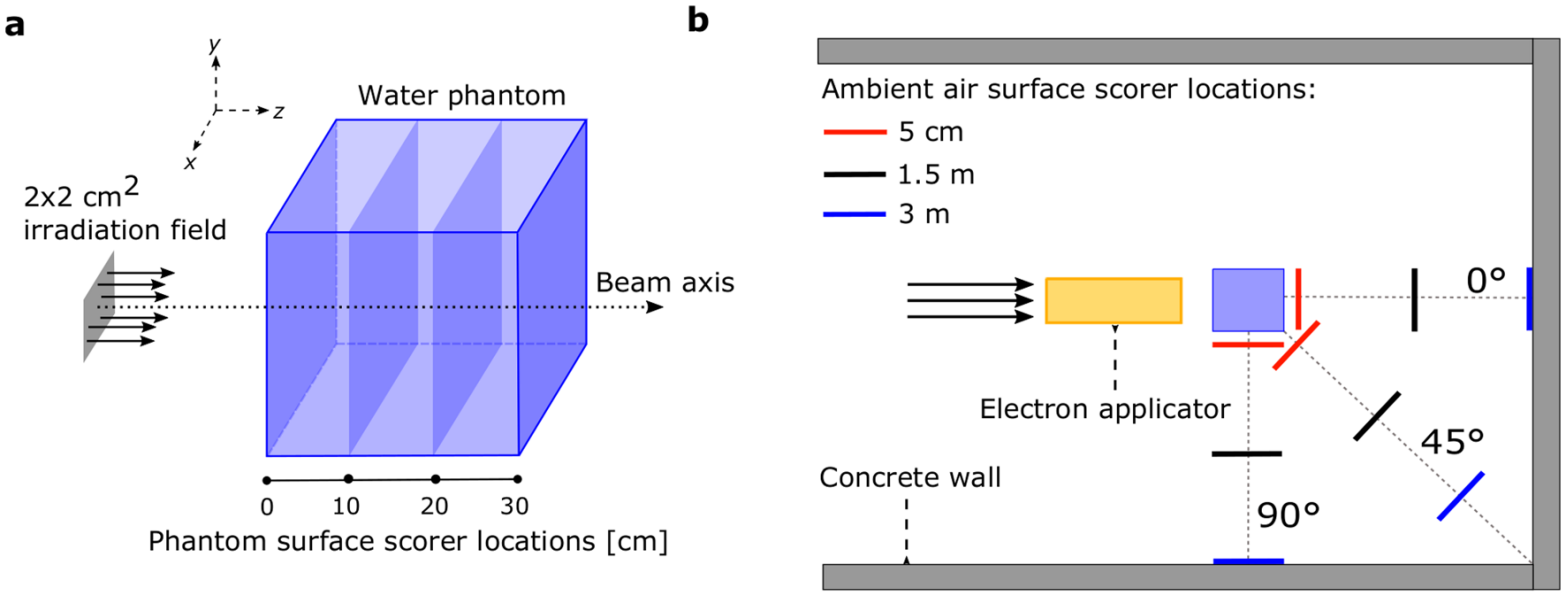
Schematic drawing of the TOPAS simulation, not drawn to scale and only for illustration purposes. **(a)** Surface scorer locations within the water phantom, and the irradiation field leaving the applicator. **(b)** Locations of all the scoring surfaces in the ambient air, along with the electron applicator, water phantom, and semi-infinite concrete walls.

The applicator was placed at a distance of 5 cm from a 30 $\times$ 30 $\times$ 30 cm$^3$ water phantom in order to emulate the air gap present between the end of the applicator and the patient’s skin in clinical treatments. The simulation configuration for a VHEE beam with a 5 cm SSD therefore represents placing this source at the exit of the applicator. This configuration was chosen so as to quantify the contribution of both the scatterings in the applicator and in the preceding 95 cm of air. All geometrical components of the simulation were then surrounded by a semi-infinite concrete wall, composed of *G4_CONCRETE* with a density of 2.3 g/cm$^3$, which is a predefined compound imported inside of Geant4 from the NIST database^29^. Ordinary concrete of this density is the most common shielding material in external beam radiotherapy treatment rooms^44^. Scorers were placed at distances of 5 cm, 1.5 m, and 3 m from the phantom, and at angles of 0$^\circ$, 45$^\circ$, and 90$^\circ$ from the central beam axis. The geometrical configuration of the simulation is displayed in Fig. 1 and details about the scorers are given in the following sections.

#### Absorbed dose and fluence scorers

Three separate avenues were chosen through which to evaluate the absorbed dose in the phantom. Firstly, PDD curves were calculated in order to have a better understanding of the behaviour in depth of these VHEE beams. This was achieved through the application of the *DoseToMedium* discretized volume scorer of TOPAS. Doses and corresponding standard deviations were scored in voxels of 5 $\times$ 5 $\times$ 1 mm$^3$ (*x, y, z*) along the central axis of the beam. These total on-axis doses were then compared against two quantities: the Bremsstrahlung contribution originating solely from the Cerrobend insert, which was scored by filtering to only include those particles or ancestors which underwent a Bremsstrahlung interaction within the Cerrobend volume, and the contribution of electrons (both primary and secondary) having undergone an interaction within the entirety of the applicator structure. Secondly, lateral dose profiles were calculated, and the collimating effect was compared against the uncollimated configuration with the use of another discretized volume scorer, with voxels of 0.5 $\times$ 5 $\times$ 5 mm$^3$. A smaller source was used for the lateral dose profiles (highlighted in Table 1) in order to gain in computation time. And thirdly, a similar volume scorer was used to evaluate the dose deposited in a 2$\times$2$\times$2 cm$^3$ target centred at a depth of 10 cm in the water phantom. The objective was not only to use the dose deposited in the target to corroborate the PDD curves, but also to evaluate the composition of the irradiation field, i.e. the relative contribution of secondary particles in relation to the simulation configuration. This was achieved by filtering according to the particle’s name and generation.

Surface scorers of 30 $\times$ 30 cm$^2$ were placed at the locations depicted in Fig. 1, which lay on the same *y*-plane as the water phantom. The particle fluences were scored with the use of TOPAS’s *SurfaceTrackCount* surface scorer, which bins the incident particles according to their energy. The scored particles were filtered according to their name in order to separate the different types of particles. Additionally, they were filtered according to the volume from which they originated in order to evaluate the contribution originating from specific components in the simulation. Python scripts were then used to perform the post-processing analysis of all these raw TOPAS outputs in the water phantom in order to obtain the relevant figures.

In order to estimate the type B uncertainty involved in the scoring of neutron fluences (and by extension the neutron yields), we investigated the consequences arising from a change of the underlying physics options governing the photonuclear processes. These processes are handled by the hadronic options of the chosen physics list^35^. While BERT was used in this study, comparisons were also made with BIC and INCLXX (Liège Intranuclear Cascade Model)—an experimental model where one of the possible applications is radioprotection considerations in the vicinity of high energy accelerators^45^. In another study of VHEEs using MC simulations, comparisons were made between the results obtained using TOPAS (Geant4) physics options, FLUKA physics options, and experimental measurements. While a 2% variation in dose distributions was observed between TOPAS and FLUKA, a 5 to 10% difference was observed between TOPAS and the experimental measurements for various beam spreads^32^. These initial indications as to the type B uncertainties for VHEE beams were used as a baseline, and increased to the conservative estimate of 20% type B uncertainty to account for differences in the simulation geometry and physics options, which was then applied to all neutron yields reported in this work.

#### Ambient neutron dose equivalent scorer

In order to quantify the effect that ionising radiation has on the human body, the ICRP defined protection quantities (organ absorbed dose, equivalent dose, effective dose) to act as limitation and optimisation guidelines^46^. Given the difficulty at measuring certain protection quantities, operational quantities were originally defined in ICRU reports 39^47^ and 43^48^ to provide estimates for the related protection quantity. The enhanced biological effectiveness and highly penetrating nature of neutrons are a concern, and given their capability to scatter throughout the treatment room, stray neutrons could potentially reach the patient and deposit an unwanted dose. Therefore area monitoring operational quantities such as H$^*$(d), which acts as an estimate of the effective neutron dose, have often been used to evaluate the degree of the presence of neutrons at various locations in a treatment room for both conventional photon and proton treatments^49,50^. It is defined as the dose equivalent at a point in a radiation field that would be produced by the corresponding expanded and aligned field in the ICRU sphere at a depth, d, on the radius opposing the direction of the aligned field, with 10 mm being the recommended depth to consider for penetrating radiations^36^.

An evaluation of this quantity within the treatment room provides indications as to which areas would be prime candidates for more advanced shielding, if indeed the neutron dose to the patient is deemed problematic. There is precedent for the use of TOPAS to calculate these dose equivalents as studies have been previously carried out for proton beams^51^. Furthermore, dose equivalent calculations using the *QGSP_BERT_HP* physics list in Geant4 have been found to be in good agreement with the dose equivalent scorers of MCNP^52^. Sets of fluence to dose equivalent conversion coefficients were recommended in ICRP publication 74^53^ and ICRU report 57^54^, and thus the ambient neutron dose equivalent can be calculated according to Eq. (1) where $\Phi _i$ is the neutron fluence for the i$^{th}$ energy bin, and $h^*(10)_i$ is the corresponding fluence to dose equivalent conversion coefficient for that energy bin^51^.

1 $$\begin{aligned} H^*(10) = \sum ^n_{i=1}h^*(10)_i\times \Phi _i. \end{aligned}$$

Calculation of this ambient neutron dose equivalent was done in TOPAS by filtering for neutrons, and making use of the *AmbientDoseEquivalent* scorer in conjunction with the *Sum* reporting option in order to perform a summation over all the energy bins of the fluence. TOPAS makes use of the conversion coefficients calculated by Pelliccioni^36^, which were made available to service new and emerging radiotherapy treatment modalities which require conversion coefficients covering higher energies than those defined in ICRP publication 74^53^ and ICRU report 57^54^. Even more recently, ICRU report 95^37^ recommended an alternative definition of the ambient dose equivalent and provided even more up to date conversion coefficients.

As detailed above, the fluence of neutrons represented our first source of type B uncertainties. Given that the ambient neutron dose equivalent is also dependent on the conversion coefficients chosen, we considered these very coefficients to be the second source of type B uncertainties. Ambient dose equivalent MC studies on MCNPX and FLUKA have been performed in which the variation induced by the use of older/newer conversion coefficients was found to be 15%^55^ and 30%^56^ respectively. Correspondingly, a conservative estimate of 30% type B uncertainty was applied to the ambient neutron dose equivalent values calculated from the default TOPAS coefficients in this study. These values were then compared against the results obtained after repeating the simulations using the newer coefficients of ICRU report 95^37^. All ambient dose equivalent simulations were performed using the BERT model. It should be noted, however, that the objective of this study was not necessarily to optimize the choice of conversion coefficient, or fine-tune the ambient neutron dose equivalent calculations of VHEEs. Rather, its importance will lie in the comparison to established treatment modalities such as proton therapy, for which the bulk of these studies were based on the older conversion coefficients.

## Results and discussion

### Absorbed dose and fluences in the water phantom

**Figure 2:**
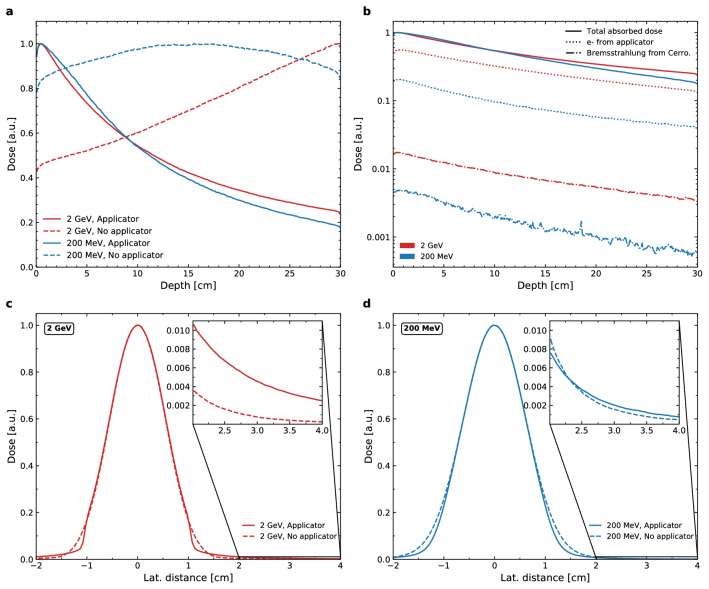
Distal and lateral dose profiles in the water phantom. On-axis PDD curves are displayed in the upper row of panels and were calculated using source 1 of Table 1. **(a)** Depicts the relative PDD curves with (solid lines) and without (dashed lines) an applicator, and for both energies (2 GeV in red and 200 MeV in blue). **(b)** Displays the relative contribution of electrons originating from the applicator structure or electrons with ancestors which originated in the applicator structure (dotted lines), the relative contribution of dose depositions due to the Bremsstrahlung interaction within the Cerrobend (dash-dot lines), and the total absorbed dose along the PDD (solid lines). The 2 GeV and 200 MeV beams are represented by the colours red and blue respectively. The bottom row of panels depicts the lateral dose profiles at a 10 cm depth in the water phantom, calculated using source 2 of Table 1. The solid line indicates the presence of an applicator, and the dashed line indicates its absence. **(c)** Contains the 2 GeV profiles, while **(d)** contains the 200 MeV profiles.

The initial investigation into the absorbed dose in the water phantom aimed to look at the nature of dose depositions in depth, with and without the electron applicator, while maintaining the same SSD of 100 cm. This behaviour in depth is depicted by the PDD curves of Fig. 2. Uncertainties on the absolute absorbed dose in each voxel along these PDD curves was maintained below 1% for both the 2 GeV and 200 MeV beams. The lateral dose profiles in Fig. 2 were scored at a depth of 10 cm in the water phantom. Similarly to the PDD curves, the statistical uncertainties on the absolute absorbed dose in each voxel of these lateral dose profiles was well below 1%, and was not able to be visualised on the curves. In order to better compare the different configurations, the relative dose was plotted. A value of 1 a.u. corresponds to the voxel with the maximum dose deposited for that specific configuration.

What we observe is that in panel a of Fig. 2, the use of an applicator results in high entrance doses (expanded upon in the following subsection), that decreases with depth into the phantom. This is true for both the 2 GeV and 200 MeV beam. When an applicator is not used, the 2 GeV profile undergoes a steady increase of dose deposited with depth, whereas the curve for the 200 MeV electrons is nearly uniform in nature. We observe that the use of an applicator drastically alters the nature of dose depositions in depth for the VHEE beams. In clinical electron beams, interactions within the head of the accelerator and collimating material both contribute to the absorbed dose. These relative contributions were evaluated in panel b of Fig. 2 and it was found that for the 2 GeV beam in particular, approximately 50% of the on-axis dose depositions are as a result of electron interactions within the applicator structure, while the contribution due to the Bremsstrahlung interaction exclusively within the Cerrobend is on the order of magnitude of a few percent. As expected, the contribution of these secondaries is reduced for the lower energy 200 MeV beam. While Cerrobend blocks are known to have a higher Bremsstrahlung contribution than tubular applicators^7^, we observe that this contribution is nevertheless still negligible when compared to the total absorbed dose.

The lateral dose profiles in the bottom row of panels of Fig. 2 highlight the effect that a Cerrobend block has—in particular on the distant penumbra region (> 1 cm lateral distance) of these profiles. For both the 2 GeV and 200 MeV profiles there is very little reduction in beam penumbra when a collimator is added, but we do observe a divergence between the two configurations occurring at approximately 1 cm lateral distance. This is expected and corresponds to the width of the Cerrobend opening. In the beginning of this distant penumbra region, the relative dose of the configuration with an applicator is lower than when an applicator is not used. However this trend is short-lived, as highlighted by the zoomed inserts in both panels a and b. For the 2 GeV beam the use of an applicator results in higher tail doses after a lateral distance of approximately 1.5 cm, which can be attributed to the scatterings within the Cerrobend. This same increase is observed for the 200 MeV profile, although the effect is not as severe. Based on these results the use of an applicator has a limited effect on the penumbra of these VHEE beams, and in the case of the 2 GeV beam there is a penalisation for its use in the form of additional dose in the tails. It should be noted however, that there is greater dosimetric importance to the slight reduction in relative dose in the distant penumbra region compared to the tails, as the tail doses correspond to less than 1% of the maximum dose.

**Figure 3:**
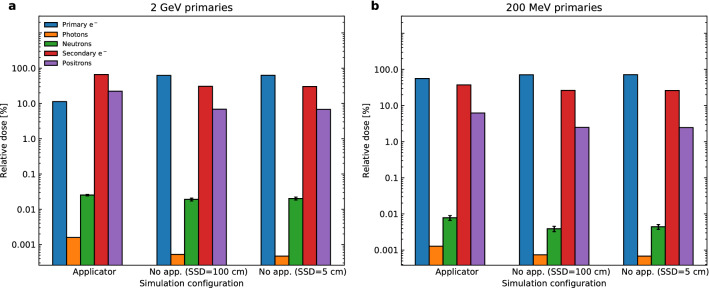
Absorbed dose contributions of secondaries to the target, as a percentage of the total target dose which was normalised to 2 Gy. Contributions of primary and secondary electrons, photons, neutrons and positrons were investigated for **(a)** 2 GeV and **(b)** 200 MeV primaries. The corresponding simulation configuration is given on the x-axis of each graph, and the percentage contribution to the 2 Gy total is given on the y-axis.

As introduced earlier, the absorbed dose in a 2 $\times$ 2 $\times$ 2 cm$^3$ target situated at a depth of 10 cm was scored, and the dose in the target was normalised to a 2 Gy total absorbed dose in order to make comparisons between each configuration. The secondaries were scored by filtering to only include the dose deposited by a specific particle. As neutrons are only indirectly ionising (due to their lack of charge), the term ‘neutron dose’ will henceforth be used to describe to dose resulting from all the secondaries produced due to neutron interactions with matter. Uncertainties on dose deposited for primary and secondary electrons, photons, and positrons were all below 1% and thus the error bars are not able to be visualised. The neutron dose, however, was subject to an uncertainty of between 4 and 17% and these error bars can be seen in Fig. 3. For the 2 GeV beam, we see that there is a considerably greater proportion of dose deposited due to photons and secondary electrons when an applicator is used compared to when an applicator isn’t used. Additionally, due to the increased presence of photons, there is a corresponding increase in dose deposited by positrons due to the pair production interaction. Moreover, a decrease in the relative contribution of the dose due to primary electrons when an applicator is used was observed. This is to be expected since a portion of the primary electrons are blocked by the collimating Cerrobend. A small percentage of the total dose deposited for a 2 GeV VHEE beam has also been attributed to the creation of muon pairs by high energy photons^22^. The dose depositions by muons and other exotic particles were included in the calculation of the total absorbed dose but were not displayed in Fig. 3. It was found that both the 2 GeV and 200 MeV beam received $\sim$ 50% higher absorbed dose due to neutrons when an applicator was used relative to when an applicator was not used.

#### Fluences and neutron yield in the water phantom

**Figure 4:**
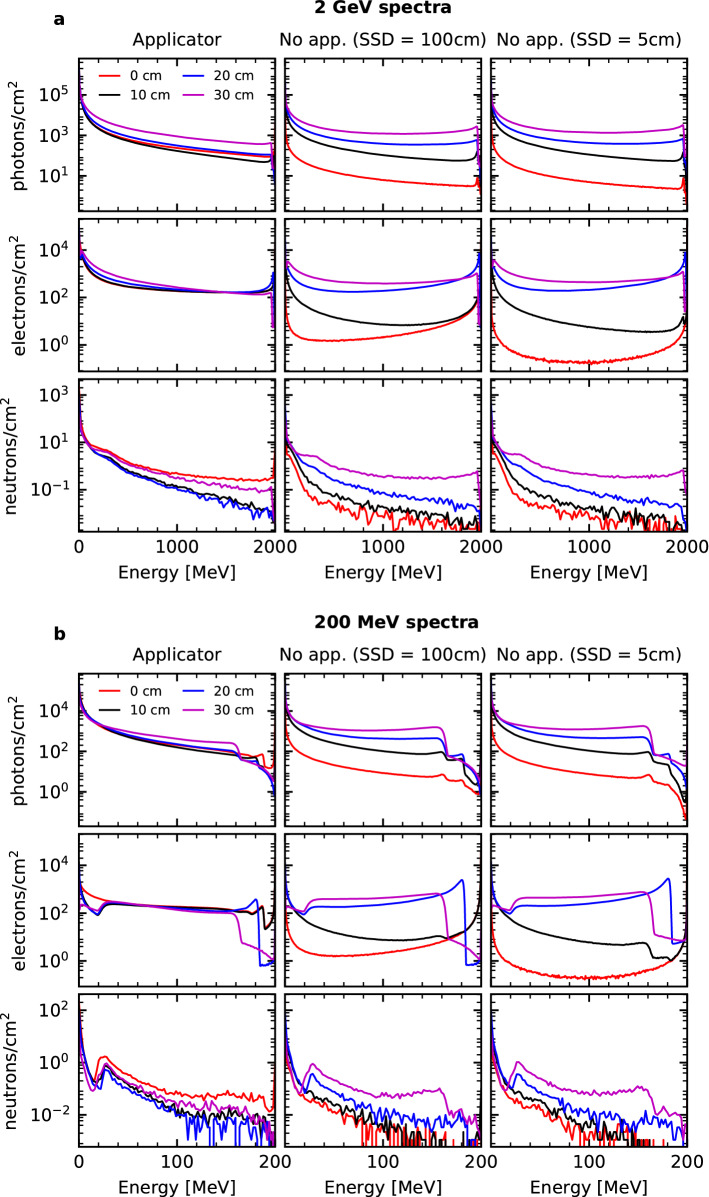
Particle fluences at 0, 10, 20, and 30 cm depth (red, black, blue, and purple curves respectively) in the water phantom for the **(a)** 2 GeV and **(b)** 200 MeV electron primaries. Each row: fluence for each particle (photons, electrons, and neutrons) in particles/cm$^2$. Each column: the corresponding simulation configuration.

Figure 4 depicts the various particle fluences resulting from the 2 GeV primaries for each simulation configuration. The fluence of positrons was not included as their behaviour is similar to that of electrons. We observe a higher yield of photons compared to electrons, which can be attributed to the Bremsstrahlung interaction which is the dominant interaction process for high energy primary electrons. The critical energy (the energy at which the Bremsstrahlung interaction begins dominating over collisional energy losses) is a few tens of MeV^57^. There is a decreasing trend in both photon and neutron fluences with increasing energy, and for electrons there exists a spike in the fluence at the 2 GeV maximum at the surface, which reduces in severity as the depth increases. At 0 cm this spike can be attributed to the 2 GeV primaries which have yet to undergo an interaction. Naturally, as the depth increases the presence of these 2 GeV electrons decreases as they undergo collisional and radiative energy losses. Regardless of the configuration evaluated, at a depth of 30 cm there is a depression in the fluence of electrons in the region of energies just below the energy of the primary particles. We observe that at a depth of 20 cm, we still maintain a spike in the fluence for 2 GeV electrons, whereas for the 200 MeV spectra, there is already a reduction in the fluence of electrons at the higher energy range. This can be attributed to the fact that the 2 GeV beams have a high inertia and are incredibly penetrating in nature—meaning that for greater depths in the water phantom, the 2 GeV beams have a higher proportion of electrons close/equal in energy to the primary electrons than for the 200 MeV beams.

What is more interesting however, is that there is a drastic drop in the fluence for all particles at shallow depths when going from the simulation configuration with an applicator to one without an applicator. This phenomenon was explained by evaluating the total fluence of particles at 0 cm compared to the fluence where a filter was applied to exclude particles (or particles with ancestors) not originating from the applicator. These fluences can be found in Supplementary Fig. S1. What was observed was that for the neutrons and photons, essentially the totality of the measured fluence at 0 cm was due to particles originating directly from an interaction within the applicator, or the descendants of the aforementioned particle. Naturally this was not the case for electrons as the Cerrobend opening allowed electrons to reach the phantom without first interacting with the applicator. Therefore this explains the drop in photon and neutron fluences in Fig. 4 when the applicator is removed. An alternative formulation of this conclusion is that the presence of an electron applicator greatly increase the photon and neutron fluence in the entrance region of the water phantom. It acts to normalise the particle fluences and make them more homogeneous in depth, i.e. it essentially eliminates whatever build-up region which may have been present.

The behaviour of the fluences in depth for both the 2 GeV and 200 MeV beams without an applicator are consistent with the results reported by Subiel et al.^24^ who evaluated the neutron fluence inside and around a water phantom for 165 MeV electron beams. The results of their MC simulations showed a quasi-isotropic neutron fluence (consistent with earlier studies^8^) with a slightly lower fluence in the first few centimetres of the phantom. The neutron yield/cm$^2$ per primary electron can be found by integrating the curves of Fig. 4 and dividing by the total number of primary electrons simulated. In this work, for the 2 GeV beam with an applicator the neutron yield/cm$^2$ per primary electron varied from approximately 3 $\times$ 10$^{-5}$ at 0 cm to 3 $\times$ 10$^{-6}$ at 30 cm. Without the applicator (100 cm and 5 cm SSD) the yield varied between approximately 5 $\times$ 10$^{-7}$ and 2 $\times$ 10$^{-6}$ for the same distances. For the 200 MeV beam with an applicator the yield fluctuated between 3 $\times$ 10$^{-6}$ at 0 cm to 2 $\times$ 10$^{-7}$ at 30 cm. Without the applicator (100 cm and 5 cm SSD) the yield was between 7 $\times$ 10$^{-8}$ and 2 $\times$ 10$^{-7}$, once again for the same distances. What we observe is that the neutron yield for the 2 GeV beam is approximately one order of magnitude higher than the 200 MeV beam. Once again the elimination of the build up region is highlighted by the decreasing trend of yield in depth whenever the applicator is used, compared to the increasing trend in the absence of the applicator. These results are consistent with Subiel et al. who observed a neutron yield in a water phantom between 10$^{-5}$ and 10$^{-7}$ neutrons/cm$^2$ per primary electron for a 165 MeV VHEE beam^24^. Supplementary Fig. S3 highlights the differences observed in the neutron yield within a water phantom depending on the physics list used. Good agreement between BERT, BIC, and INCLXX was observed with all data points lying within the 20% type B uncertainty estimate.

Given what has been discussed above, this section can be summarised as follows. For both the 2 GeV and 200 MeV beams the effect of adding a collimating structure in the form of an applicator works to significantly reduce the depth of the dose build-up region, resulting in a depth dose profile which is considerable at shallow depths, and falls to approximately 20% of the maximum dose at a depth of 30 cm. The lateral dose profiles highlighted the slight reduction in distant beam penumbra (> 1 cm lateral distance) with the addition of a collimating Cerrobend block, and the penalisation in the tails of the 2 GeV profile due to scatterings therein. Furthermore the neutron yields for the 2 GeV beam were one order of magnitude higher than for the 200 MeV beam, and in all cases were almost two orders of magnitude higher in the entrance region when an applicator was used compared to when no applicator was present. One of the limitations of this study however, is that fluences were only evaluated at specific locations in the water phantom and thus only gross inter-surface trends are visible. More comprehensive conclusions would be able to be drawn if a fluence map was instead created. Regardless, either similar or lower neutron yields were obtained in this work compared to other studies of VHEEs^8,22,24^ which all concluded that there is likely no significant additional adverse effect of neutrons in VHEE treatments compared to conventional treatments. Therefore, we can conclude that the addition of a collimating material in the path of these VHEE beams does not lead to an enhancement of the neutron fluence that is considerably different from the values calculated in these aforementioned studies.

### Particle yields and dose equivalent in ambient air

#### Neutron yield in ambient air

**Figure 5:**
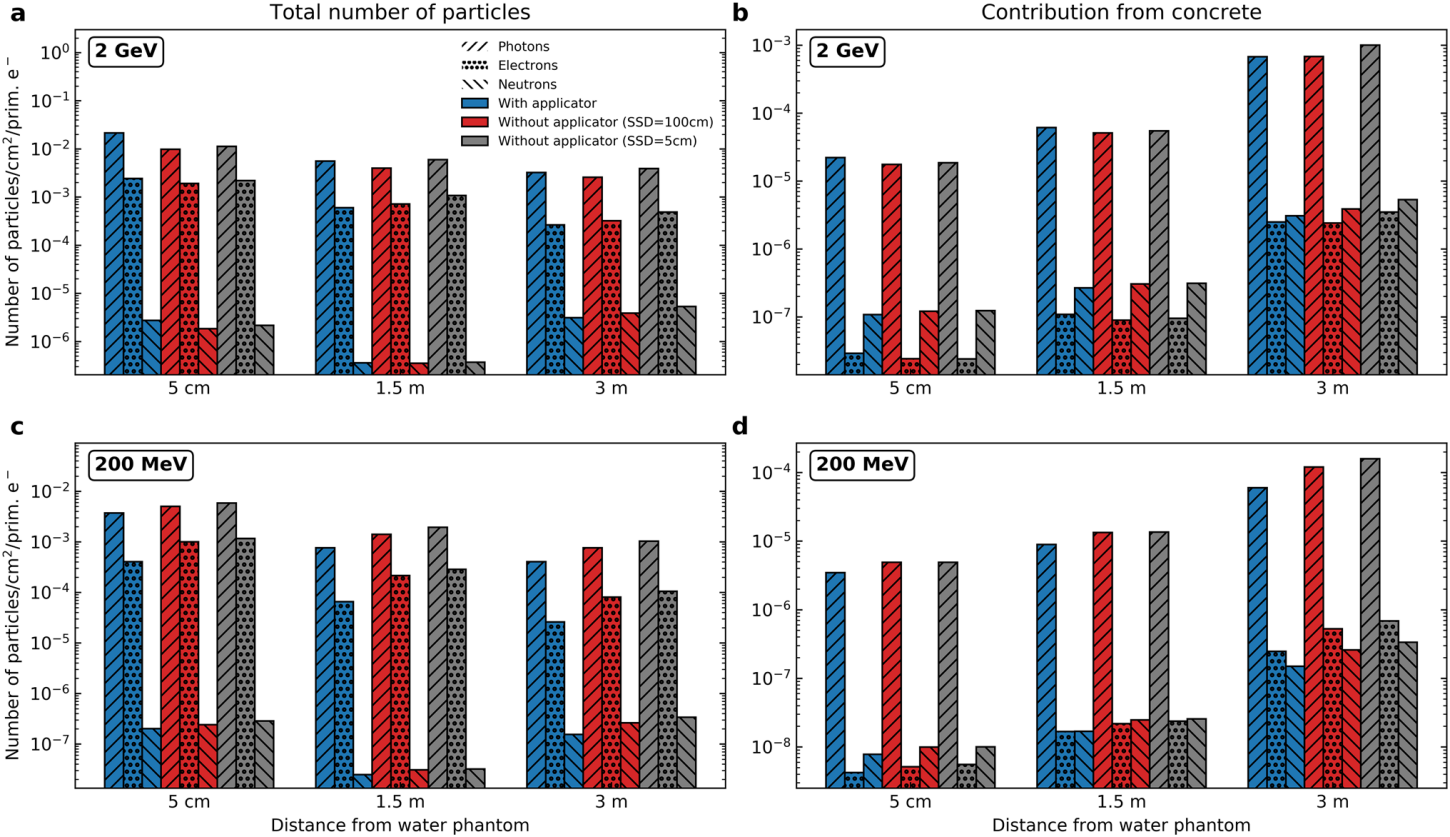
Particle yields in number of particles/cm$^2$/primary electron for all distances and configurations at 0$^\circ$, and for both the 2 GeV and 200 MeV beams. Each configuration is represented by a colour, and each particle is represented by a different hatching. Upper row: 2 GeV primaries. Bottom row: 200 MeV primaries. Total yields from all sources is given in **(a,c)**, while the yield contribution due to the concrete walls is given in **(b,d)**.

The previous sections evaluated the absorbed dose, particle fluences, and neutron yield within the water phantom. The remainder of this study looks at the ambient air, exterior to the water phantom and surrounded by semi-infinite concrete walls. As detailed in the “Materials and methods” section, TOPAS surface scorers were used to record and bin the particle fluences at various distances and off-axis angles from the water phantom. These fluences in isolation did not reveal any noteworthy trends, unlike the particle fluences in the water phantom. Indeed this is to be expected, given that in the previous section the only major change elucidated was the fact that the applicator has an effect primarily in the entrance region of the phantom. What was more interesting was the calculation of the total yields resulting from these fluences, and comparing them with the yields arising only from particles which originated in the concrete wall, along with their corresponding descendants. Figure 5 depicts this very comparison for both the 2 GeV and 200 MeV beams at 0$^\circ$ and for all configurations. Each configuration is given a different colour, and each particle a different bar hatching.

Looking first at the total yields, the behaviour for photons and electrons is as expected. Regardless of the configuration or particle energy, the highest yield of these particles is found at the 5 cm distance and decreases with increasing distance from the water phantom. No substantial conclusions are able to be drawn vis-à-vis inter-configuration effects since the differences between each configuration were not substantial. The trend of decreasing particle yield with distance, however, is not replicated by the neutrons. For both the 2 GeV and 200 MeV beams, there appears to be a reduction in the neutron yield from 5 cm to 1.5 m, followed by an increase in the yield from 1.5 m to 3 m. This increasing characteristic of the neutron yield can be attributed to the presence of a concrete wall—as depicted by the second column of panels in Fig. 5. For this case, the highest neutron yield for both the 2 GeV and 200 MeV beams was found at 3 m, i.e. right next to the concrete wall, and decreased towards the water phantom. What this highlights is that the presence of the concrete wall acts to inject additional neutrons into the treatment room near the wall’s vicinity, thus counteracting the otherwise decreasing yield with increasing depth from the phantom. Furthermore, while the total yield is dominated by photons and electrons, the composition of the radiation field is different for the yield coming from the concrete. Photons are still the dominant particle, however the neutron and electron yields are similar at 3 m. There is a decreasing trend for all particle yields coming from the wall for distances closer to the phantom, nevertheless the neutron yield dominates over the electron yield at 5 cm, particularly for the 2 GeV beam.

For the 2 GeV beam, the total neutron yield was on the order of approximately 10$^{-6}$ neutrons/cm$^2$/primary electron, while for the 200 MeV beam, this yield was approximately one order of magnitude lower. Considering that these values are similar to those found for the neutron yields for their respective energies in the water phantom being irradiated, an investigation into the ambient neutron dose equivalent - with particular attention paid to the values at 3 m—is warranted and outlined in the following section. The variation of neutron yield in the ambient air depending on the physics list is depicted in Supplementary Fig. S4. The largest differences were observed for the neutron yield calculated using INCLXX. Nevertheless, a conservative estimate of 20% type B uncertainty was sufficient to account for all variations of the absolute neutron yield due to changes in physics options.

#### Ambient neutron dose equivalent

**Figure 6:**
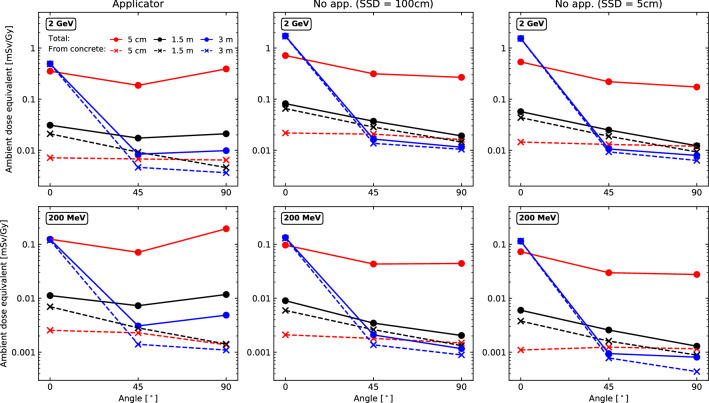
Ambient neutron dose equivalent per treatment gray at 5 cm, 1.5 m, and 3 m from the water phantom, for angles of 0$^\circ$, 45$^\circ$, and 90$^\circ$ from the central beam axis, and for all simulation configurations. Upper row: 2 GeV primaries. Bottom row: 200 MeV primaries. Each column represents a specific configuration, and each colour represents a distance from the water phantom. Solid lines with circular markers are the total dose equivalent values, while dashed lines with cross markers are dose equivalent values based on the fluence of neutrons coming from the concrete.

As summarised in the “Materials and methods” section, the ambient neutron dose equivalent is found from the product of the neutron fluence with specific fluence to dose equivalent conversion coefficients^36^. This dose equivalent was evaluated for all simulation configurations, all distances, and all angles. In order to make comparisons between each configuration, the ambient neutron dose equivalent was normalised with the dose delivered, D, to the 2 $\times$ 2 $\times$ 2 cm$^3$ target in the water phantom. Therefore the quantities reported in Fig. 6 are $\frac{H^*(10)}{D}$ values, with units of mSv/treatment Gy.

Error bars are excluded from Fig. 6 in order to facilitate the visualisation of the results. All statistical uncertainties for the total ambient neutron dose equivalent were maintained below 12%, with the lowest being 0.28% (2 GeV electrons, with applicator, at 0$^\circ$ and 300 cm) and the highest being 11.48% (200 MeV electrons, no applicator with a 100 cm SSD, at 90$^\circ$ and 300 cm). Supplementary Figure S5 and Supplementary Table S1 depict the variation in ambient dose equivalent depending on the conversion coefficients used, in which a 30% type B uncertainty estimate was found to be a sufficiently conservative estimate. As highlighted earlier, the combined uncertainty was calculated by performing a summation in quadrature of the type A uncertainty (statistical uncertainty) and type B uncertainty (arising due to changes in physics options and conversion coefficients). These combined uncertainties are shown in Table 2.

The total ambient neutron dose equivalent is depicted by the solid lines in Fig. 6. With the exception of the 200 MeV beam with an applicator, the highest values are generally found at 0$^\circ$ and 3 m, i.e. right besides the concrete wall, as inferred by the particle yield graphs of Fig. 5. This is a consequence of the photons that were forward scattered in the water phantom. At this same angle, the second highest dose equivalent occurs at 5 cm and the lowest is found at 1.5 m. At 45$^\circ$ and 90$^\circ$ the highest total dose equivalent is always found at 5 cm, followed by the dose equivalent at 1.5 m and finally 3 m. Once again, we consider this a consequence of the forward scatter photons in the water phantom. Given its proximity, the 1.5 m location is more susceptible to be exposed to the majority of neutrons which appear to be travelling along the beam axis (at 0$^\circ$). Consequently there is a lower contribution from concrete at larger angles. The 2 GeV electron primaries experienced a maximum total ambient neutron dose equivalent of approximately 1.717 ± 0.619 mSv/Gy while the maximum value for the 200 MeV primaries was found to be 0.1942 ± 0.0701 mSv/Gy. Generally, the total ambient neutron dose equivalent for the 200 MeV primaries is one order of magnitude lower than for the 2 GeV beam.

For both electron energies, all distances from the water phantom, and all configurations, as the angle increases from 0$^\circ$ to 45$^\circ$ there is a decrease in the dose equivalent. What is interesting is that for the configurations in which an applicator is absent, the dose equivalent is either maintained at approximately the same level or decreases between 45$^\circ$ and 90$^\circ$. This is in contrast to the configuration with an applicator, where the dose equivalent is either maintained at the same level or increases between 45$^\circ$ and 90$^\circ$. This seems to indicate a broader scattering of the neutrons due to the electron applicator. Additionally, for the applicator configurations, there is a more severe increase between 45$^\circ$ and 90$^\circ$ for the 200 MeV beam as opposed to the 2 GeV beam which could be attributed to the broader photon scattering angles for the lower energy electron beam.

The dashed lines of Fig. 6 depict the ambient dose equivalent arising from the neutrons originating from interactions in the concrete wall, or neutrons whose ancestors originated in the wall. For the configurations without an applicator, and for both electron energies, there is close agreement between the total dose equivalent values at 1.5 m and 3 m, and the respective values of the dose equivalent contribution from concrete. Thus indicating that the majority of the dose equivalent at these distances is due to the concrete’s contribution to the neutron fluence, with the smallest difference occurring at 3 m and 0$^\circ$. Similar conclusions were reached for the increase in neutron dose equivalent in the vicinity of concrete walls for proton beams^56,58^. Considerable differences between the total and the contribution from concrete are observed at 5 cm, suggesting the dominance of neutrons from the water phantom at this distance. The two aforementioned statements hold true for the configurations with an applicator, however, for the dose equivalent values at 1.5 m and 3 m there is an increase in difference between the total values and the contribution from concrete between 45$^\circ$ and 90$^\circ$. This highlights and adds credence to the previous hypothesis that due to the presence of an applicator neutrons are more broadly distributed, resulting in an increase in the difference between the total dose equivalent and the dose equivalent due to the contribution from the concrete. The ranges of the ambient neutron dose equivalent values for each configuration can be found in Table 2, within which comparisons to dose equivalent studies of proton beams were made.

**Table 2:** Range of ambient neutron dose equivalent values for this work with a combined uncertainty (statistical type A, and 20% and 30% type B for variations due to physics options and conversion coefficients respectively), compared with other studies involving protons.

References	Particle	Simulation details/treatment modality	Neutron dose equivalent [mSv/Gy]	Details for range of dose equivalent values
This work	2 GeV, VHEE	With applicator	0.0084 ± 0.0031 to 0.491 ± 0.177	At 300 cm 45$^\circ$ to 0$^\circ$
		Without applicator (SSD = 100 cm)	0.0115 ± 0.0042 to 1.717 ± 0.619	At 300 cm 90$^\circ$ to 0$^\circ$
		Without applicator (SSD = 5 cm)	0.0079 ± 0.0029 to 1.538 ± 0.555	At 300 cm 90$^\circ$ to 0$^\circ$
	200 MeV, VHEE	With applicator	0.0031 ± 0.0011 to 0.1942 ± 0.0701	At 300 cm 45$^\circ$ to 5 cm 90$^\circ$
		Without applicator (SSD = 100 cm)	0.0012 ± 0.0005 to 0.1333 ± 0.0481	At 300 cm 90$^\circ$ to 0$^\circ$
		Without applicator (SSD = 5 cm)	0.0008 ± 0.0003 to 0.1142 ± 0.0412	At 300 cm 90$^\circ$ to 0$^\circ$
Schneider et al.^56^	Protons	Spot scanned pencil beam	0.02 to 7	177 MeV beam. Dose equivalent from 100 cm to 5 cm (lateral distances from central beam axis)
Charyyev et al.^51^		With a minibeam collimator	0.017 to 3.23	120 MeV beam. Dose equivalent from 0$^\circ$, 105 cm from phantom to 135$^\circ$ and 11 cm from water phantom
		Uncollimated pencil beam	0.0013 to 0.242	120 MeV beam. Dose equivalent from 135$^\circ$, 105 cm from phantom to 0$^\circ$ and 11 cm from water phantom
Zheng et al.^55^		Passively scattered	0.3 to 19	100 MeV, 14.1 cm diameter scattered field to 250 MeV, 35.4 cm diameter scattered field

The last column of the table provides details about each value in the dose equivalent range, such as the location of the calculation/measurement and beam characteristics.

The studies by Zheng et al.^55^ and Charyyev et al.^51^ are MC studies carried out on MCNPX and TOPAS respectively. These MC calculations have concluded, similarly to experimental studies^56,59,60^, that ambient neutron dose equivalent values of a few mSv per treatment gray can be expected—depending on the size of the field, presence/absence of a physical collimator, and beam energy among other physical parameters. These results are corroborated by the review paper by Hälg et al.^58^, discussing neutron dose measurements in proton therapy. The primary conclusions reached were that the neutron dose in proton therapy is unlikely to have a considerable influence on the risk of secondary cancers. Furthermore, the ambient neutron dose equivalent in active scanning treatments is lower compared to passively scattered protons, and in general, is also lower than the neutron dose equivalents in the vicinity of the patient for conventional RT treatments with photons, which lie in the range of approximately 0.1 to 20.4 mSv/Gy^61^.

Table 2 provides preliminary indications that a clinical implementation of VHEEs would be quite similar to conventional proton therapy treatments, given the ambient neutron dose equivalent values of a couple of mSv per treatment gray. Although there have been more published works showing the benefits of VHEEs in the 200 MeV energy range compared to 2 GeV beams, we show that from an ambient neutron dose equivalent point of view there should be no considerable radioprotection issues outside of the norm for even higher energy electron beams when compared to conventional treatments. The increase in dose equivalent in the vicinity of the concrete wall does however, warrant extra precautions be taken to ensure it is maintained below an acceptable level. The highest dose equivalent observed in this study was approximately 1.7 mSv/Gy for the uncollimated 2 GeV electron beam at 0$^\circ$ and 300 cm from the water phantom. Although the same beam in the presence of a collimating applicator yielded a lower neutron dose equivalent at this location, the results indicate that when an applicator is used one can expect a broader scattering of the neutrons, and higher dose equivalents near the collimating structure.

One of the limitations of this study is that the scoring surfaces used in the calculation of the neutron dose equivalent were placed at a limited amount of locations. This gives a general idea of the area monitoring considerations one would need to take into account, however a full picture would only be able to be gleamed by considerably expanding the locations investigated. Secondly, the induced activation caused by secondary neutrons was not considered. Although previous studies^8,22,24^ have concluded that the induced radioactivity is negligible in terms of its contribution to the dose deposited within a water phantom, it would nevertheless be interesting to evaluate the induced radioactivity originating from both the collimating structure and the concrete wall.

## Conclusion

The most notable challenges to the clinical implementation of VHEEs lay in the impracticality of producing such beams in existing medical LINACs, the increasing entrance and exit doses for increasing electron energies, and the concern of excessive secondary neutron production. While these challenges have been systematically tackled over recent years, in this study the aspects we investigated were the dosimetry and radioprotection issues within a treatment room of both collimated and uncollimated VHEE beams. We calculated the resulting out-of-field ambient neutron dose equivalents in the treatment room allowing us to better situate VHEE beams compared to clinical photon/proton beams.

The neutron yield in ambient air appeared to initially decrease with distance from the water phantom, but increased in the vicinity of the concrete wall due to neutrons originating from the interactions therein. The highest ambient neutron dose equivalent was found to be approximately 1.7 mSv/Gy for the 2 GeV beam, with the 200 MeV beam yielding values one order of magnitude lower. Thus the neutron dose equivalent with VHEEs can be considered comparable to that of conventional proton therapy treatments, and even in the worst case scenario—when there is an additional physical collimation of the beam in the form of an applicator—the level of neutron production in all cases will not require additional shielding. We observed that the presence of an applicator acts to considerably reduce the depth of the dose build-up region. Therefore the trade-off for lower exit doses is a much higher entrance dose—this being particularly severe for the 2 GeV beam compared to the 200 MeV beam. Without an applicator, there is a gradual increase in the yield of neutrons in the phantom with increasing depth, however adding an applicator acts to homogenize this yield resulting in significantly more neutrons in the phantom’s entrance region. Despite this, the highest neutron yield was found for the 2 GeV beam with an applicator to be on the order of 10$^{-5}$ neutrons/cm$^2$ per primary electron, and is therefore similar to other studies which concluded no additional adverse effects of neutrons compared to conventional RT treatments.

The prevailing sentiment amongst leading experts is that the future of VHEEs for clinical treatments is contingent on the successful clinical implementation of compact laser wakefield accelerators with sufficient shot-to-shot stability, which enables the delivery of these high energy beams using magnetic collimation. While this magnetic collimation is inconsistent with the use of an electron applicator, we investigated whether or not these collimated beams may still be meritocratic in terms of its influence on the dose distribution. We noted that the addition of a collimating Cerrobend had little impact in reducing the beam penumbra, and while a reduction was observed at the start of the distant penumbra region, additional dose was deposited in the tails—particularly for the 2 GeV beam. Nevertheless, the doses are still comparable and these characteristics would need to be weighed up against its advantages—such as the additional space made available due to the lack of a magnetic scanning system, and the comparative inexpensiveness of the technology.

We conclude that the use of an applicator to more precisely target the tumor results in additional neutrons in the first few centimetres of the water phantom, as well as a broader scattering of those neutrons in the ambient air surrounding the collimating structure. Furthermore, given the relatively low ambient neutron dose equivalent, a clinical implementation of collimated or uncollimated VHEEs would likely not warrant any supplementary safeguards from a radioprotection point of view compared to current conventional RT treatments, which therefore facilitates the clinical translation of this novel therapeutic modality.

## Supplementary Information

Supplementary Information.

## Data Availability

All data associated with the work published in this paper will be made available from the corresponding author upon request.
