# Supplementary material for: Dosimetry and radioprotection evaluations of very high energy electron beams
Source: Sci Rep. 2021 Oct 12;11:20184. doi: 10.1038/s41598-021-99645-7 (PMC8511248; doi:10.1038/s41598-021-99645-7)
Supplement: Supplementary file 1 — Supplementary Information. [file 41598_2021_99645_MOESM1_ESM.pdf]

Dosimetry and radioprotection evaluations of very high energy electron beams

Thongchai A. M. Masilela[1,2], **Rachel Delorme**[3], **and Yolanda Prezado**[1,2,*]

[1]Institut Curie, Université PSL, CNRS UMR3347, Inserm U1021, Signalisation radiobiologie et cancer, 91400, Orsay, France
[2]Université Paris-Saclay, CNRS UMR3347, Inserm U1021, Signalisation radiobiologie et cancer, 91400, Orsay, France
[3]Univ. Grenoble Alpes, CNRS, Grenoble INP, LPSC-IN2P3, 38000 Grenoble, France
[*]yolanda.prezado@curie.fr

Supplementary material

Fluences and neutron yield in the water phantom

As elucidated in the main text, we observe a drop in the fluences at shallow depths when going from the simulation configuration with an applicator to one without an applicator. One of the avenues through which to evaluate the effect of this applicator on the resulting particle fluences within a water phantom was to apply the *OnlyIncludeIfParticleOrAncestorFromVolume* filter in TOPAS. The resulting particle fluences are depicted in Supplementary Fig. S1.

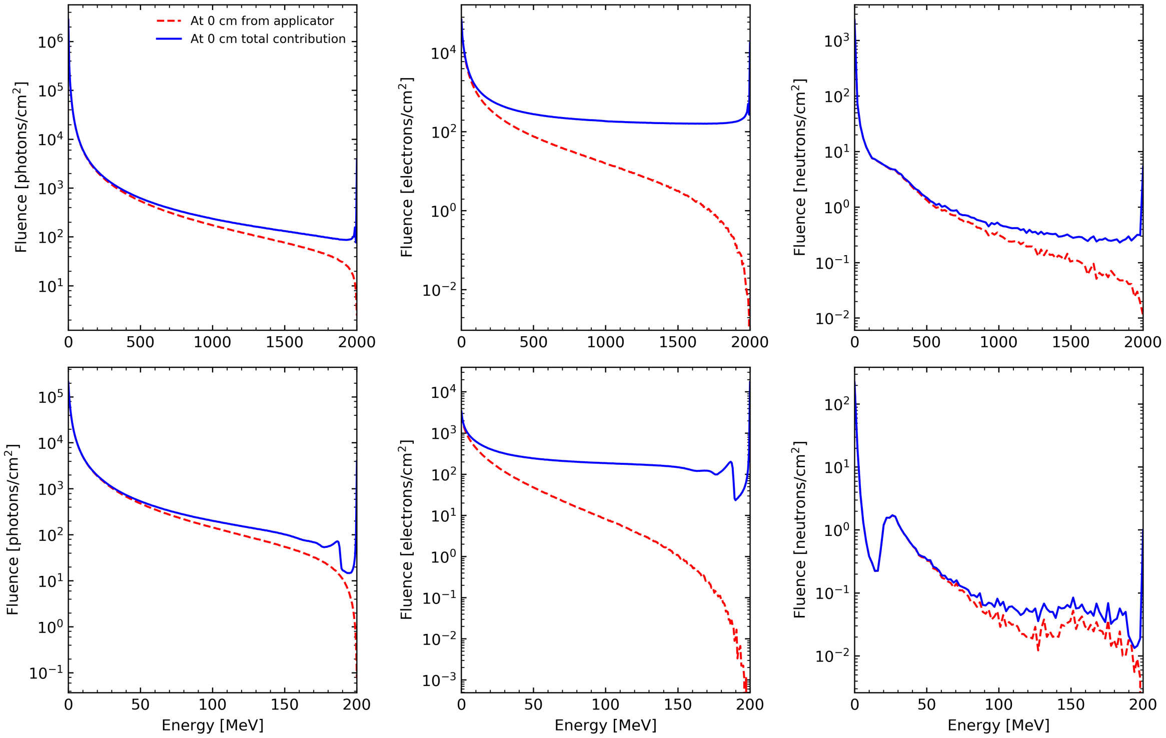

Supplementary Figure S1. Particle fluences at 0 cm in the water phantom, with the solid blue line indicating the total contribution (i.e. all particles of that type crossing the 0 cm boundary), and the dashed red line indicating only those particles directly originating from, or with ancestors originating from, the components of the applicator. Upper row: 2 GeV primaries. Bottom row: 200 MeV primaries. First, second, and third columns: photons, electrons, and neutrons respectively.

Supplementary Figure S1 highlights, and identifies, the use of an applicator as the reason for the drop in neutron and photon fluences at shallow depths when an applicator is removed. For the neutrons and photons, a majority of the calculated fluence is

a result of particles originating directly from an interaction within the applicator, or descendants of the aforementioned particles. Resultingly, when an applicator is removed we observe the aforementioned fluence drop at 0 cm. Contrastingly, a majority of the low energy electrons originate from the applicator, and as the energy increases, the area between the two fluences can then be said to be comprised of primary electrons, electrons that have lost their energy in air before reaching the phantom, and electrons that have been backscattered in the phantom.

In order to aid in the visualisation of how the particle yields change with distance into the water phantom, the bar graph of Supplementary Fig. S2 was created. What this figure makes evident is that there is an increase in the yield of photons, electrons, and neutrons when an applicator is used. Furthermore, while the neutron yield in the absence of an applicator is largely increasing with increasing distance into the water phantom, the opposite becomes true when an applicator is added. There is a clear injection of a large amount of neutrons in the first few centimetres of the phantom due to the presence of the applicator. These inter-configuration differences in the yield of neutrons appear to be normalised at 30 cm into the phantom, giving more credence to the conclusion reached in the main text that the presence of an applicator only appears to be consequential in its immediate vicinity.

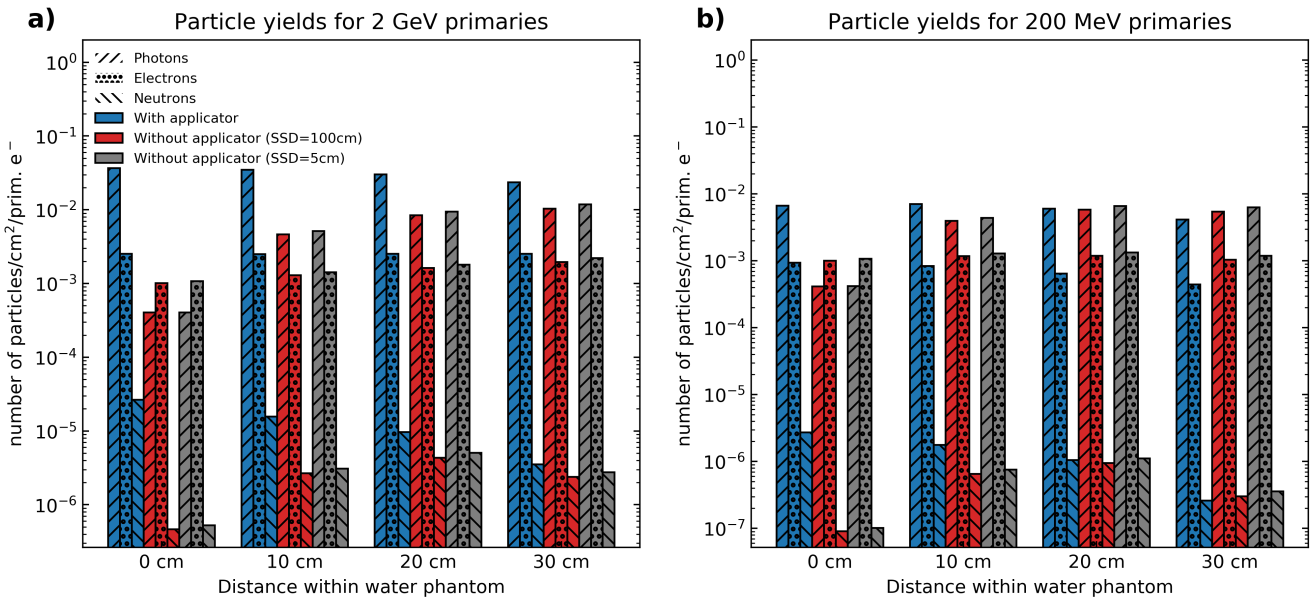

Supplementary Figure S2. Particle yields in number of particles/cm^2/primary electron for 0, 10, 20, and 30 cm in the water phantom for both the 2 GeV and 200 MeV beams. Each configuration is represented by a colour, and each particle is represented by a different bar hatching. Total yields for the 2 GeV primaries are given in panel **a)** while total yields for the 200 MeV primaries are given in panel **b)**

Type B uncertainty estimates

As highlighted in the main text, the estimation of the type B uncertainty was performed through two avenues. Firstly, the underlying physics options governing the photonuclear process (responsible for the production of neutrons) was modified from the BERT model to BIC and INCLXX[1]. Secondly, newer sets of conversion coefficients were used from which the ambient neutron dose equivalent was calculated. Supplementary Figure S3 depicts the variation in neutron yield within the water phantom for different physics options. The error bars correspond to the type B uncertainty estimate of 20%, applied to the original results (BERT) that were displayed in the main text.

We observed that for the 2 GeV beam, all data points for both BIC and INCLXX were within 5% of the BERT data. This same conclusion was reached for the 200 MeV beam BIC data points. This, however, was not the case for the 200 MeV INCLXX data which had some points with a greater than 5% difference - the largest being approximately 12% for the configuration without an applicator for a 5 cm SSD at 0 cm in the water phantom. Nevertheless all data points were comfortably within the 20% type B uncertainty estimate applied to the BERT results - indicating that this conservative estimate should be sufficient in accounting for the variations in neutron production as a result of different physics options. The same analysis was performed for the fluence of neutrons in the ambient air, as visualised in Supplementary Fig. S4.

Similarly to the results for neutron yield within the water phantom, there was good agreement between the BERT and BIC/INCLXX models for the 2 GeV beam, with all points for the total yield being within 5% while the yield contribution due

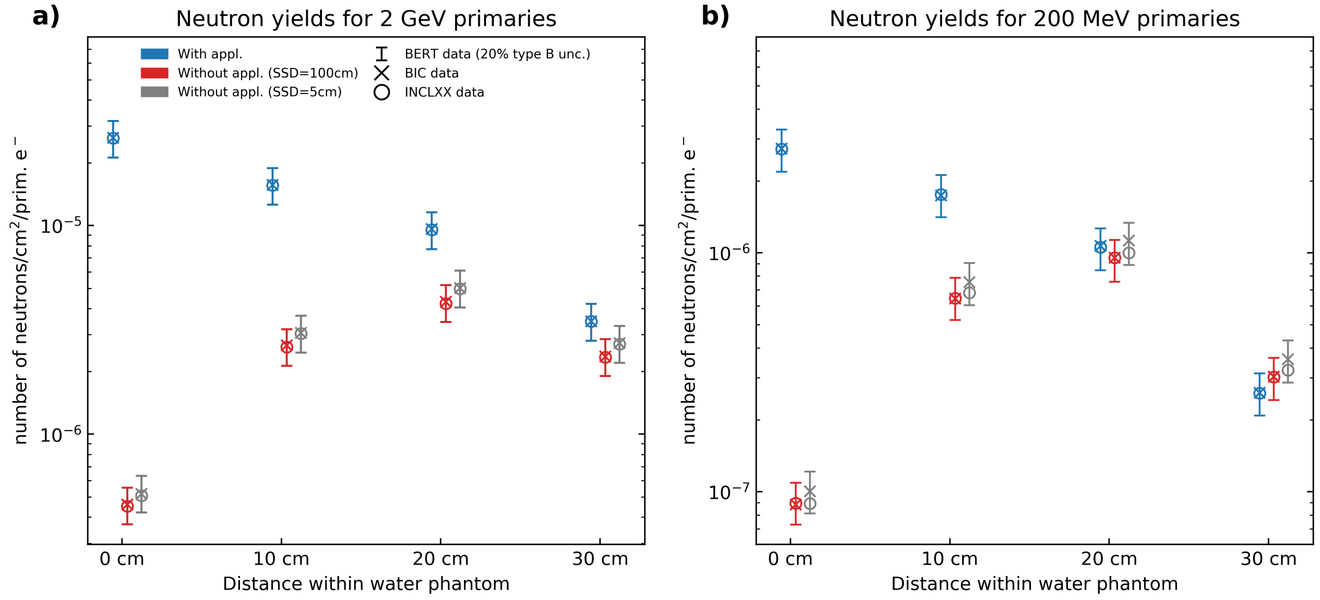

Supplementary Figure S3. Neutron yields in number of neutrons/cm^2/primary electron for 0, 10, 20, and 30 cm in the water phantom for both the **a)** 2 GeV and **b)** 200 MeV beams. The 20% type B uncertainty applied to the BERT results shown in the main text are depicted by the error bars, the BIC and INCLXX results are marked with a cross and circle respectively, and each configuration is allocated a different colour.

to concrete was within 8%. All the BIC data points for the 200 MeV beam were also maintained below this 8% threshold, while the INCLXX model displayed a greater variation, reaching a maximum of approximately 17% for the total neutron yield without an applicator for a 5 cm SSD at a distance of 1.5 m from the water phantom. These results indicated that neutron yield in both the water phantom and ambient air were more susceptible to a change from BERT to INCLXX than from BERT to BIC. Nevertheless, it was found that the 20% type B uncertainty estimate was sufficient to account for possible variations in physics options.

The second avenue through which the type B uncertainty was estimated was through a variation of the conversion coefficients used to calculate the ambient neutron dose equivalent. Supplementary Figure S5 depicts the differences in the total ambient neutron dose equivalent arising when the default coefficients used in TOPAS[2] were changed to those described ICRU report 95[3]. As indicated in the main text, an estimate of 30% type B uncertainty was applied to the original results obtained using the default TOPAS coefficients. This 30% uncertainty is depicted by the error bars in the Supplementary Fig. S5.

Barring one outlier for the 200 MeV beam (without an applicator for an SSD of 100 cm at 3 m from the water phantom at an angle of 45°) all variations in ambient neutron dose equivalent due to a change in the conversion coefficient were within the 30% estimate. The 20% type B estimate for the neutron yield and the 30% estimate for the change of conversion coefficients were combined in quadrature with the statistical uncertainty to yield a combined uncertainty. Table 2 from the main text was reproduced below with an additional column added to indicate the percentage difference observed in the ambient neutron dose equivalent values reported, when changing from the default TOPAS coefficients to those recommended in ICRU report 95.

As highlighted in Supplementary Table S1, not only are the largest dose equivalent values for each configuration within the 30% estimate, but they also represent an overestimation as compared to the dose equivalent values obtained using the newer coefficients - thus ensuring that the results reported, and corresponding conclusions, are based on conservative estimates.

References

1. Mancusi, D. *et al.* Extension of the liège intranuclear-cascade model to reactions induced by light nuclei. *Phys. Rev. C* **90**, 054602, DOI: https://doi.org/10.1103/PhysRevC.90.054602 (2014).

2. Pelliccioni, M. Overview of Fluence-to-Effective Dose and Fluence-to-Ambient Dose Equivalent Conversion Coefficients for High Energy Radiation Calculated Using the FLUKA Code. *Radiat. Prot. Dosim.* **88**, 279–297, DOI: https://doi.org/10.1093/oxfordjournals.rpd.a033046 (2000).

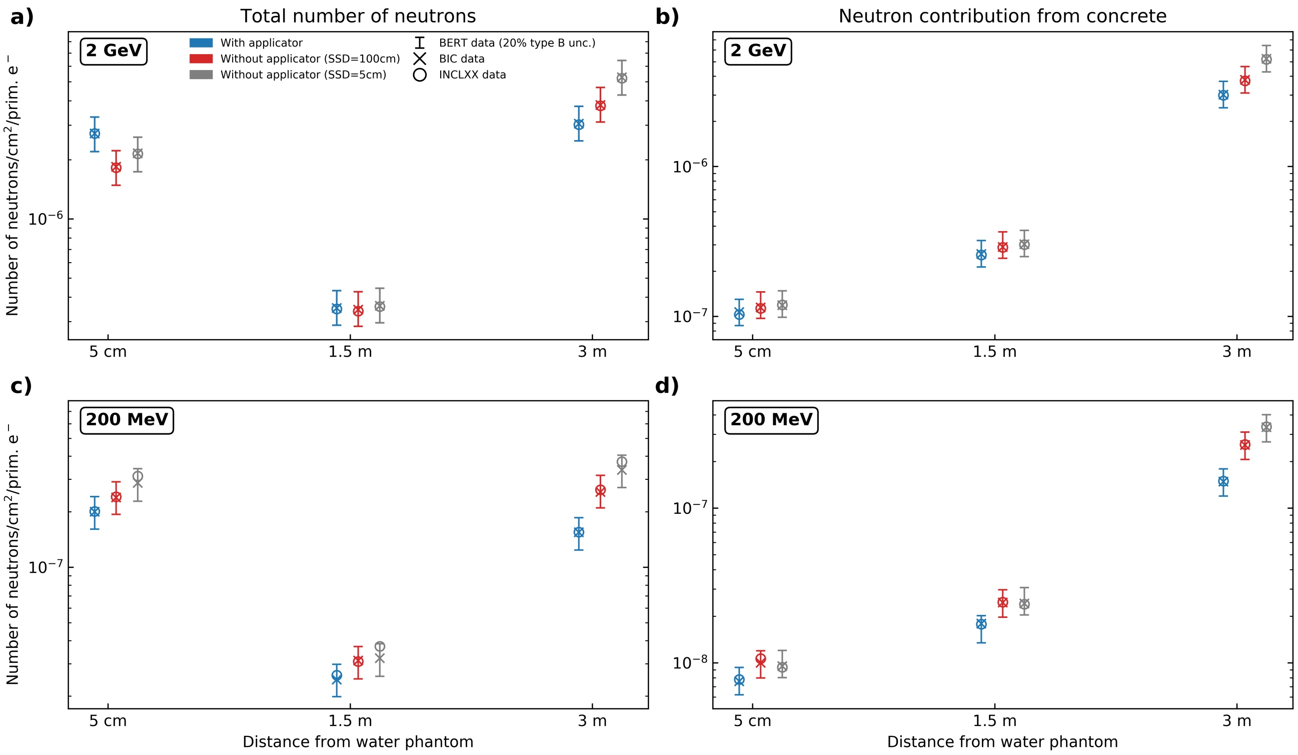

Supplementary Figure S4. Neutron yields in number of neutrons/cm^2/primary electron for all distances and all configurations at 0°, for both the 2 GeV and 200 MeV beams. The 20% type B uncertainty applied to the BERT results shown in the main text are depicted by the error bars, the BIC and INCLXX results are marked with a cross and circle respectively, and each configuration is allocated a different colour. Panels **a)** and **c)** depicts the total neutron yield, while panels **b)** and **d)** depict the neutron yield contribution due to the concrete walls.

3. International Commission on Radiation Units and Measurements. 4 Conversion coefficients. *J. ICRU* **20**, 30–37, DOI: https://doi.org/10.1177/1473669120966214 (2020).

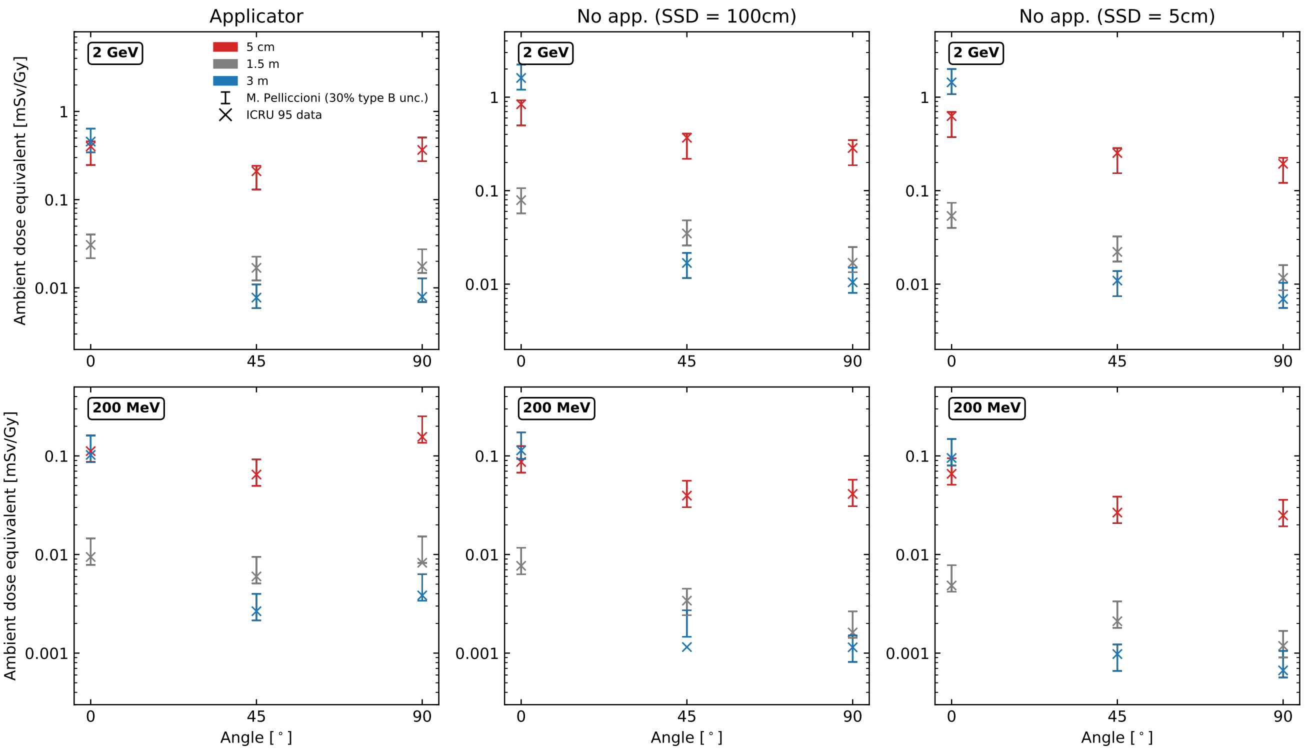

Supplementary Figure S5. Total ambient neutron dose equivalent per treatment gray at 5 cm, 1.5 m, and 3 m from the water phantom, for angles of 0°, 45°, and 90° from the central beam axis, and for all simulation configurations. The upper row of panels depicts the results for the 2 GeV beams, while the 200 MeV beam results are shown in the bottom row of panels. Each column represents a different configuration. Error bars are indicative of the 30% type B uncertainty applied to the ambient neutron dose equivalent values obtained using the default TOPAS coefficients. The cross markers are representative of the values obtained using the coefficients reported in ICRU report 95.

Beam energy	Simulation details / treatment modality	Neutron dose equivalent [mSv/Gy]	Locations in air for range of values	TOPAS to ICRU 95 coefficients [% difference]
2 GeV	With applicator	0.0084 ± 0.0031 to 0.491 ± 0.177	At 300 cm. 45 ° to 0°	-7.8% to -19.8%
	Without applicator (SSD = 100 cm)	0.0115 ± 0.0042 to 1.717 ± 0.619	At 300 cm. 90° to 0°	-9.2% to -6.5%
	Without applicator (SSD = 5 cm)	0.0079 ± 0.0029 to 1.538 ± 0.555	At 300 cm. 90° to 0°	-12.7% to -6.4%
200 MeV	With applicator	0.0031 ± 0.0011 to 0.1942 ± 0.0701	At 300 cm 45° to 5 cm 90°	-13.3% to -19.7%
	Without applicator (SSD = 100 cm)	0.0012 ± 0.0005 to 0.1333 ± 0.0481	At 300 cm. 90° to 0°	-1.4% to -14.7%
	Without applicator (SSD = 5 cm)	0.0008 ± 0.0003 to 0.1142 ± 0.0412	At 300 cm. 90° to 0°	-17.4% to -16.6%

Supplementary Table S1. Range of ambient neutron dose equivalent values for this work with a combined uncertainty (statistical type A, and 20% and 30% type B for variations due to physics options and conversion coefficients respectively). The last column of the table indicates the percentage difference of dose equivalent range reported, when the ICRU 95 conversion coefficients are used in place of the default TOPAS coefficients.
